# Antiproteinuric effect of dapagliflozin attenuates tubule-interstitial injury development in subclinical acute kidney injury animal model

**DOI:** 10.3389/fphar.2026.1899334

**Published:** 2026-09-01

**Authors:** Rodrigo A. S. Peres, Ana Carolina Pinto-Figueiredo, Douglas E. Teixeira, Rodrigo P. Silva-Aguiar, Sarah A. S. Alves, Giulianne Bastos Serpa, Carlos P. Gomes, Wagner Barbosa Dias, Ana Acacia S. Pinheiro, Celso Caruso-Neves

**Affiliations:** 1 Carlos Chagas Filho Institute of Biophysics, Federal University of Rio de Janeiro, Rio de Janeiro, Brazil; 2 National Institute of Advanced Technologies in Diagnosis and Prognosis of Chronic and Neglected Diseases, INATEC, Rio de Janeiro, Brazil; 3 Clementino Fraga Filho University Hospital, Federal University of Rio de Janeiro, Rio de Janeiro, Brazil; 4 Rio de Janeiro Innovation Network in Nanosystems for Health-NanoSAÚDE/FAPERJ, Rio de Janeiro, Brazil

**Keywords:** dapagliflozin, proteinuria, proximal tubule, SGLT2I, subAKI, tubule-interstitial injury

## Abstract

**Introduction:**

Subclinical acute kidney injury (subAKI) is characterized by markers of tubular injury without changes in glomerular function, being associated with acute kidney injury and chronic kidney disease. The use of dapagliflozin (Dapa) has shown protective effects in different models of kidney diseases. However, its effects on the development of subAKI are still under investigation. Previous results suggested that dapagliflozin effect on proximal tubule epithelial cells may involve protein *O*-GlcNAcylation. Therefore, we aimed to investigate the effects of dapagliflozin on subAKI and its associated mechanisms.

**Methods:**

Male BALB/c mice (6–8-week-old) were separated in four experimental groups: control (CTL), subAKI, subAKI + Dapa, and Dapa. The subAKI mouse model was induced by albumin overload for 7 days. Treated animals received dapagliflozin (1.0 mg/kg/day) by oral gavage. Renal functional and structural parameters were analyzed. *In vitro* experiments were performed in LLC-PK1 to test specific effects of dapagliflozin on PTECs.

**Results:**

Glomerular structure and function were preserved across all experimental groups. However, subAKI animals exhibited increased albuminuria, urinary β2-microglobulin excretion, and elevated KIM-1 expression, all of which were attenuated by dapagliflozin treatment. In addition, dapagliflozin restored albumin-FITC uptake in the renal cortex of subAKI animals. Consistent with these findings, dapagliflozin prevented the reduction in fluorescein isothiocyanate-conjugated albumin (albumin-FITC) uptake induced by high albumin concentration (20 mg/mL) in LLC-PK1 cells. Furthermore, both (Na^+^+K^+^)ATPase activity and α1-subunit expression were reduced in the subAKI and subAKI + Dapa groups. To investigate the mechanisms underlying the effects of dapagliflozin, we evaluated the expression of OGA, OGT, and total protein *O*-GlcNAcylation in the renal cortex. Notably, the increases in OGT expression and total protein *O*-GlcNAcylation observed in subAKI animals were significantly attenuated by dapagliflozin treatment.

**Conclusion:**

These results suggest that the protective effect of dapagliflozin on tubule-interstitial injury development in subAKI is associated with the restoration of total protein *O*-GlcNAcylation balance, thereby contributing to its anti-proteinuric effect. These results indicate that dapagliflozin could be a possible alternative to the treatment of subAKI.

## 1 Introduction

Kidney diseases are an important burden on the health system and patients worldwide (Eckardt et al., 2013). The progressive and silent feature of kidney diseases increases the risk for the development of acute kidney injury (AKI) and the progression to chronic kidney disease (CKD) (Romagnani et al., 2017; Kellum et al., 2021). It is well recognized that albuminuria is a hallmark in kidney disease being a biomarker and a risk factor for progression to end-stage kidney disease (Heerspink et al., 2026). Protein overload in the proximal tubule (PT) induces tubule-interstitial injury (TII), acting as an active contributor to the development and/or progression of kidney disease (Molitoris et al., 2022).

Currently, the diagnosis of AKI, based on KDIGO guidelines, relies on an increase in plasma creatinine and/or a decrease in urinary output (KDIGO Work Group, 2012). However, due to the renal functional reserve (RFR), a decline in glomerular function may only manifest when more than 50% of renal function is already compromised (Ronco et al., 2017). In this context, the use of tubular injury markers has enabled the identification of a syndrome known as subclinical acute kidney injury (subAKI). This condition is characterized by evidence of structural or cellular tubular damage in the absence of overt changes in glomerular function parameters (Haase et al., 2012; Dépret et al., 2020).

SubAKI has been identified as a risk factor for the development of AKI and CKD, being associated with worsening clinical outcomes (Haase et al., 2012; Dépret et al., 2020). The mechanism underlying the development of subAKI involves the reduction of megalin-mediated albumin endocytosis in proximal tubule epithelial cells (PTECs), which triggers a pro-inflammatory phenotype leading to the development of tubule-interstitial injury (TII) and tubular albuminuria (Peruchetti et al., 2021; Peres et al., 2023a; Liu et al., 2015). SubAKI is increasingly recognized as a therapeutic window where interventions in PTECs albumin handling could ameliorate TII development and, consequently, avoid the progression of kidney damage and risk of developing AKI or CKD (Vanmassenhove et al., 2019; Zou et al., 2022).

The use of gliflozin, a specific inhibitor of sodium-glucose cotransporter 2 (SGLT2i), has emerged as a possible anti-proteinuric agent used for the treatment of diabetic and non-diabetic CKD patients (Heerspink et al., 2019; EMPA-KIDNEY Collaborative Group, 2022). In a randomized clinical trial using dapagliflozin (DAPA-CKD), patients presented a reduced risk of kidney function decline, progression to end-stage kidney disease or death from renal or cardiovascular causes when compared to placebo (Heerspink et al., 2020). Importantly, dapagliflozin presented an anti-proteinuric effect, reducing albumin-to-creatinine ratio, which is correlated with better outcomes in kidney disease treatments (Heerspink et al., 2020). However, the mechanism underlying this process remains unclear.

Accordingly, signaling pathways that modulate albumin reabsorption in PTECs are likely to play a role in the pathogenesis of subAKI development (Peruchetti et al., 2018; Peruchetti et al., 2021; Kapetanaki et al., 2022). *O*-GlcNAcylation is a post-translational modification (PTM) that regulates protein function through the reversible addition of β-N-acetylglucosamine (GlcNAc) to serine and threonine residues (Hart et al., 2007). This dynamic PTM is controlled by two key enzymes that add and remove GlcNAc moieties, known as O-GlcNAc transferase (OGT) and O-GlcNAcase (OGA) (Hart et al., 2007). As a stress response, *O*-GlcNAcylation levels may be harmful or protective depending on the context (Fahie et al., 2022). In contrast-induced AKI, an increase in *O*-GlcNAc levels was protective against tubular damage, apoptosis, and oxidative stress (Hu et al., 2017). On the other hand, the use of OSMI-1, an inhibitor of OGT, that decreased *O*-GlcNAcylation, improved survival and glomerular function in Shiga toxin-injected mice (Lee et al., 2022).

Previously, our group demonstrated that dapagliflozin reduced total protein *O*-GlcNAcylation in LLC-PK1 cells exposed to high-glucose conditions (Silva-Aguiar et al., 2023). In addition, dapagliflozin treatment improved TII in a rat model of early-stage diabetes (Farias et al., 2023). Furthermore, in spontaneously hypertensive rats (SHR), increased *O*-GlcNAcylation levels in PTECs were associated with reduced albumin reabsorption and exacerbated TII (Silva-Aguiar et al., 2018). Based on these findings, we hypothesized that dapagliflozin treatment may represent an alternative therapeutic strategy to ameliorate subAKI syndrome.

In the present study, we aimed to investigate the effects of dapagliflozin treatment on the development of TII associated with protein handling and cortical protein *O*-GlcNAcylation. A subAKI animal model was established by daily intraperitoneal injections of bovine serum albumin (BSA) for 7 days. Our results demonstrate that dapagliflozin attenuates TII development in subAKI, a finding that was correlated with an anti-proteinuric effect and a reduction in cortical protein *O*-GlcNAcylation.

## 2 Methods

### 2.1 Materials and reagents

Bovine serum albumin (BSA) fraction V (#A9647), BSA conjugated to fluorescein isothiocyanate (BSA-FITC), phenylmethylsulfonyl fluoride (PMSF), and protease inhibitor cocktail (no. I3786), MOPS, HEPES, EDTA, sucrose, Triton X-100, Tween 20, sodium fluoride, sodium pyrophosphate, sodium orthovanadate, sodium β-glycerophosphate, tetramethylethylenediamine (TEMED), acrylamide, bromophenol blue, 2-mercaptoethanol, periodic acid-Schiff (PAS) reagent, Sirius red, Harry’s hematoxylin, Folin and Ciocalteu’s phenol reagent were purchased from Sigma-Aldrich (St. Louis, MO, United States). Polyvinylidene fluoride membranes and methanol were purchased from Merck Millipore (Barueri, SP, Brazil). ECL Prime, sodium dodecyl sulfate, and Tris were purchased from GE Healthcare (Pittsburgh, PA, United States). Dulbecco’s modified Eagle’s medium (DMEM), phosphate-buffered saline (PBS), fetal bovine serum (FBS), antibiotic-antimycotic (100×), UltraPure N,N′-methylenebisacrylamide (bisacrylamide) was purchased from Thermo Fisher Scientific (Waltham, MA, United States). The following kits were purchased from Labtest (Lagoa Santa, MG, Brazil): Albumina (ref. 19), Sensiprot (ref. 36), Creatinina (ref. 35-100), Sódio Enzimático (ref. 124), Urea CE (ref. 27), and Glicose HK Liquiform (ref. 137). Antibodies anti-rabbit IgG HRP-linked (#7074), anti-mouse IgG HRP-linked (#7076), rabbit monoclonal anti-GAPDH (14C10) (#2118) were purchased from Cell Signaling Technology (Danvers, MA, United States). Mouse monoclonal anti-TIM1 [3D9F5] (ab233720) and rabbit monoclonal anti-Albumin [EPR20195] (ab207327) were purchased from Abcam (Cambridge, MA, United States). Mouse monoclonal anti-β2 microglobulin (SAB470014), mouse monoclonal anti-Na^+^/K^+^ATPase α-1 clone C464.6 (#05-369), and rabbit anti-OGA (SAB4200311) were purchased from Sigma-Aldrich (St. Louis, MO, United States). OGT (AL-28) was obtained from Hart Laboratory (Iyer et al., 2003; Lucena et al., 2016). Mouse RL-2 antibody (anti-*O*-GlcNAc) sc-59623 was purchased from Santa Cruz (Dallas, TX, United States).

### 2.2 Animals

Male BALB/c mice, six to 8 weeks old and weighing 18–24 g, used in the present study were obtained from the Animal Care Facility of the Health Science Center at the Universidade Federal do Rio de Janeiro (UFRJ), Rio de Janeiro, Brazil. All mice were kept in an air-conditioned environment (22 °C–24 °C) in a regular 12-h light/dark cycle with water *ad libitum* and fed standard chow. All procedures were approved by the Institutional Ethics Committee of the UFRJ (CEUA 083/23) and conducted in accordance with the National Institutes of Health (NIH) Guide for the Care and Use of Laboratory Animals.

### 2.3 Subclinical AKI animal model and dapagliflozin treatment

The subAKI model was developed by albumin overload as previously described (Portella et al., 2013; Peruchetti et al., 2021; Peres et al., 2023a). Animals were randomly assigned to four experimental groups: 1) control (CTL), mice received vehicle (saline I.P and water by oral gavage); 2) subAKI, animals received bovine serum albumin (BSA) intraperitoneally at 10 g/kg/day for seven consecutive days; 3) subAKI plus dapagliflozin (subAKI + Dapa), animals received a daily dose of BSA I.P (10 g/kg/day) and a dose of dapagliflozin (1 mg/kg/day) for seven consecutive days; 4) Dapagliflozin (DAPA), mice received a daily dose of dapagliflozin alone (1 mg/kg/day) for seven consecutive days.

The dapagliflozin dose was selected based on previous studies (Hodrea et al., 2020; Cai et al., 2023) and corresponds to a human equivalent dose of approximately 0.081 mg/kg/day (≈5.7 mg/day for a 70-kg adult), calculated using the FDA-recommended body surface area normalization method (Nair and Jacob, 2016). This dose falls within the clinically approved therapeutic range for dapagliflozin (5–10 mg/day). Furthermore, pharmacokinetic studies have demonstrated that dapagliflozin administered at 1 mg/kg/day effectively induces glycosuria in mice (Tahara et al., 2016), consistent with the increased urinary glucose excretion observed in the present study. Forty-eight hours prior to the seventh day of the experimental period, all mice were individually housed in metabolic cages for urine collection and measurement of food and water intake. The animals had free access to food and water until anesthesia induction.

At the end of the experimental period, mice were deeply anesthetized with a mixture of ketamine (240 mg/kg) and xylazine (15 mg/kg) administered intraperitoneally. Following confirmation of anesthesia, blood samples were collected by cardiac puncture, and animals were euthanized by exsanguination. Kidneys were subsequently harvested for functional, molecular, and histological analyses. Blood and 24-h urine samples were used to evaluate renal function parameters. The kidneys were perfused with citrated saline for functional and molecular analysis, including *in vivo* albumin uptake. When indicated, paraformaldehyde 4% were perfused for fixation for histological analysis as previously described (Peruchetti et al., 2020; Peres et al., 2023a; Farias et al., 2023).

### 2.4 Analysis of renal function

Kidney function was assessed according to previous studies (Teixeira et al., 2019; Peruchetti et al., 2020; Peres et al., 2023a). Briefly, immediately after 24 h urine collection, all samples were centrifuged 5 times (10,000 × *g* for 10 min) to remove urine sediments. Total urine volume was used to calculate urinary flow (µL/min). Then, urinary concentration of glucose, sodium, creatinine, total proteins, and albumin were measured. The plasma was obtained by centrifuging whole blood (2,500 × *g* for 5 min) followed by analysis of glucose, sodium, creatinine and blood urea nitrogen (BUN) concentrations. All urinary and plasma analysis were performed by using commercial kits from Labtest (Lagoa Santa, MG, Brazil).

Urinary concentration of total proteins and albumin were divided by urinary creatinine concentration to determine the urinary protein-to-creatinine ratio (UPCr) and urinary albumin-to-creatinine ratio (UACr), respectively. The clearance of creatinine, albumin, glucose and Na^+^ were calculated using the following equation:

$${\text{Clearance}}_{\left(\text{solute}\right)}=\frac{\left[U_{\left(\text{solute}\right)}\right]\times \text{Urine}\;\text{Flow}\;\text{Rate}}{\left[P_{\left(\text{solute}\right)}\right]}$$

The creatinine clearance was used to estimate GFR (mL/min). The fractional excretion (FE) of albumin, glucose, and Na^+^ were calculated using the following equation:

$${\text{FE}}_{\left(\text{solute}\right)}=\left(\frac{\left[U_{\left(\text{solute}\right)}]\times [P_{\text{Creatinine}}\right]}{\left[P_{\left(\text{solute}\right)}]\times [U_{\text{Creatinine}}\right]}\right)\times 100$$

Where [U_(solute)_] is the urinary concentration of the solute of interest, [P_(solute)_] is the plasma concentration of the solute of interest, and [P_Creatinine_] and [U_Creatinine_] represent the plasma and urinary creatinine concentrations, respectively.

### 2.5 Histological analysis

Histological analysis was conducted as previously described (Peruchetti et al., 2020; Peres et al., 2023a; Farias et al., 2023). Briefly, perfused kidneys were fixed in 10% buffered formalin and embedded in paraffin. Kidney sections of 5 μm and 8 µm thickness were stained with PAS and Picrosirius red, respectively. Histological analyses were performed in four animals per group, which were randomly selected to minimize potential selection bias. When no statistically significant differences were observed, *post hoc* power analyses were performed based on the observed data variability to determine whether increasing the sample size would be necessary to detect a biologically meaningful effect. The results of these analyses guided our decision on whether additional animals should be included for each experiment.

Images of the renal cortex were captured using a Nikon Eclipse 80i microscope (Nikon, Japan). Image quantification was performed in a blinded manner using Image-Pro Plus Software (Media Cybernetics, Rockville, MD, United States). The cortical tubule-interstitial area was analyzed by directly measuring the space between cortical tubules and expressed as a percentage of the total area. Tubular injury score was determined semi quantitatively as previously described by analyzing the presence of tubular injury (tubular dilation, tubular atrophy, tubular cast formation, sloughing of tubular epithelial cells or loss of brush border and thickening of tubular basement membrane) based on the following score system: score 0: no tubular injury; 1: <10% tubules injured; 2: 10 to <25% tubules injured; 3: 25 to <50% of tubules injured; 4: 50 to <75% of tubules injured; and 5: 75% or more of tubules injured (Chen et al., 2013). Interstitial cellularity was analyzed by quantifying cell nuclei in the cortical interstitial area, expressed as cells/image field. Collagen deposition was assessed by measuring the intensity of red fibers in hot spot areas and reported as a percentage of total area. The glomerular area, reported in µm^2^, was defined as the area enclosed by the outer boundary of Bowman’s capsule. Glomerular cellularity was analyzed by quantifying cell nuclei in glomerular tuft, expressed as cells/tuft area.

### 2.6 Albumin-FITC uptake *in vivo*

*In vivo* albumin-FITC uptake in the renal cortex was assessed as previously described (Peruchetti et al., 2020; Peres et al., 2023a; Peres et al., 2023b). Briefly, mice were intravenously injected with BSA-FITC (5 μg/g body weight) *via* the tail vein. The kidneys were harvested after 15 min. The renal cortex was isolated and homogenized in ice-cold Ringer solution (20 mM HEPES–Tris [pH 7.4], 5 mM D(+)-glucose, 2.7 mM KCl, 140 mM NaCl, 1 mM MgCl_2_, 1.8 mM CaCl_2_) supplemented with 1 mM PMSF and 1× protease inhibitor cocktail. Homogenates were clarified by centrifugation at 10,000 × g for 10 min. The fluorescence associated with the renal cortex was measured in the supernatant using a SpectraMax M2 microplate reader (Molecular Devices, San Jose, CA, United States). Albumin-FITC uptake was normalized to the total protein concentration of each sample and expressed as fold change of control.

### 2.7 Sample preparation and immunoblotting analysis

Kidney cortex homogenates were prepared as previously published (Peruchetti et al., 2020; Peruchetti et al., 2021; Silva-Aguiar et al., 2022a; Silva-Aguiar et al., 2022b; Peres et al., 2023a; Peres et al., 2023b). In brief, kidney cortex was carefully isolated and homogenized in ice-cold sample buffer (250 mM sucrose, 10 mM HEPES, 2 mM EDTA, 5 mM PMSF, and 1× protease inhibitor cocktail) by mechanical homogenizer followed by centrifugation for 5 times (10,000 × *g* for 10 min) for sediments removal. Homogenates were normalized by total protein concentration and prepared in 5X Laemmli buffer. Immunoblot was performed using 50 µg of total protein. The samples were resolved in 9% SDS-PAGE and transferred to PVDF membranes, blocked with 5% BSA. Then, membranes were incubated with primary antibodies overnight at 4 °C, followed by incubation with secondary antibodies at room temperature for 1 h.

Urinary samples were clarified by centrifugation 5 times (10,000 × *g* for 10 min). Urine samples were normalized according to urinary creatinine concentration. Equal amounts of urinary creatinine were loaded onto each lane, resolved by 12% SDS-PAGE, and transferred to PVDF membranes for immunoblot analysis. To analyze urinary albumin and β2M, membranes were blocked with 5% milk. ECL Prime was used for detection. Urinary protein profile was analyzed by staining SDS-PAGE gels with 0.125% Coomassie brilliant blue R-250 solution overnight at 4 °C. All the images were acquired by using the Image Quant LAS4000 Image processing system (GE Healthcare Life Sciences, Pittsburgh, PA, United States). The images were prepared and quantified with FIJI software version 2.1.0 (NIH, Bethesda, MD, United States).

### 2.8 (Na^+^+K^+^)ATPase activity

(Na^+^+K^+^)ATPase activity was determined in renal cortex homogenates as previously described (Queiroz-Madeira et al., 2010; Peruchetti et al., 2011; Arnaud-Batista et al., 2016; Silva et al., 2018; Teixeira et al., 2020). The homogenates were prepared as described above and stored at −80 °C until analysis. ATPase activity was assessed in a reaction mixture composed of 4 mM MgCl_2_, 5 mM ATP (specific activity 0.27 μCi/nmol [γ^32^P]ATP), 20 mM HEPES-Tris (pH 7.0), 120 mM NaCl, and 30 mM KCl. [γ^32^P]ATP served as a tracer to quantify inorganic phosphate (^32^Pi) release. To determine ouabain-sensitive (Na^+^+K^+^)ATPase activity, parallel reactions were performed in the presence of 1 mM ouabain. The assay was initiated by adding protein samples to achieve final concentrations of 0.5 mg/mL followed by incubation at 37 °C for 20 min. The reactions were terminated by the addition of ice-cold activated charcoal in 0.1 N HCl and centrifuged at 1,255 × g for 5 min. The supernatant was collected and released ^32^Pi was quantified using a liquid scintillation counter (Packard Tri-Carb 2100 TR). Specific (Na^+^+K^+^)ATPase activity was calculated as the difference in ^32^Pi release between assays performed in the absence and presence of ouabain. Enzyme activity is expressed as nmol Pi·mg^-1^ protein·min^-1^.

### 2.9 Cell culture and albumin-FITC uptake *in vitro*

LLC-PK1 cells, a widely used porcine PTEC cell line (Hull et al., 1976; Takakura et al., 1995; Nielsen et al., 1998) were purchased from ATCC (Rockville, MD, United States). Cells were maintained in low-glucose DMEM supplemented with 10% fetal bovine serum (FBS) and 1% penicillin/streptomycin and incubated at 37 °C in a humidified atmosphere containing 5% CO_2_, as previously described (Teixeira et al., 2019; Peruchetti et al., 2020; Peruchetti et al., 2021; Silva-Aguiar et al., 2022a; b; Peres et al., 2023a; Peres et al., 2023b). For experimental procedures, cells were plated in 24-well plates and allowed to grow for 48 h until reaching approximately 85%–90% confluence. Subsequently, cells were incubated with BSA (20 mg/mL) and/or dapagliflozin (5 μM) under serum-starved conditions overnight to evaluate albumin endocytosis *in vitro*.

The albumin uptake was conducted as previously published (Peruchetti et al., 2020; Peruchetti et al., 2021; Silva-Aguiar et al., 2022a; b; Peres et al., 2023a; Peres et al., 2023b). After the overnight treatment described above, cells were washed three times and incubated for 30 min at 37 °C with Ringer solution (20 mM HEPES–Tris, pH 7.4; 5 mM D(+)-glucose; 2.7 mM KCl; 140 mM NaCl; 1 mM MgCl_2_; 1.8 mM CaCl_2_) containing 100 μg/mL FITC-labeled bovine serum albumin (BSA-FITC). Then, plates were placed on ice and unbound BSA-FITC was removed by ten washes with ice-cold Ringer solution. The cells were lysed with a buffered solution containing MOPS (20 mM, pH 7.4) and Triton X-100 (0.1%). The lysates were collected, and fluorescence intensity was measured with a SpectraMax M2 microplate reader (Molecular Devices). Specific uptake was calculated by subtracting nonspecific fluorescence, determined in cells co-incubated with excess unlabeled BSA (100 mg/mL), from total fluorescence values. Fluorescence signals were normalized to total protein concentration in each sample, and results are presented as fold of the control group.

### 2.10 Statistical analysis

General plasma and urinary parameters were measured in all animals to confirm the successful establishment of the experimental model. However, not all analyses could be performed in the same animals because some experimental procedures were mutually exclusive. Specifically, animals used for histological analyses could not be used for SDS-PAGE, immunoblotting, or *in vivo* albumin uptake experiments because they were perfused with 4% paraformaldehyde. Likewise, animals used for *in vivo* albumin uptake studies could not be used for other analyses because they received BSA-FITC injections. As a result, the number of animals available for each analysis differed among experimental groups.

All results are presented as mean ± standard deviation (Mean ± SD). The Shapiro–Wilk test was used to assess the normality of data distribution. Group comparisons were performed using one-way analysis of variance (ANOVA), followed by Tukey’s *post hoc* test. A *p*-value <0.05 was considered statistically significant. Statistical analyses were conducted using GraphPad Prism 8 (GraphPad Software, version 8, San Diego, CA, United States).

## 3 Results

### 3.1 Dapagliflozin attenuates tubule-interstitial injury in a mouse model of subclinical acute kidney injury

The possible effects of dapagliflozin (Dapa) treatment on the development of subAKI were assessed by generating four experimental groups as detailed in the Methods Section: 1) control, CTL, 2) subclinical acute kidney injury, subAKI, 3) subAKI treated with dapagliflozin, subAKI + Dapa, and 4) control animals treated with dapagliflozin, Dapa. Basic parameters are presented in Table 1. Body weight, kidney weight–to–body weight ratio, and water intake did not differ among the experimental groups. A small but significant increase in food intake and sodium intake was observed in the Dapa group. However, plasma sodium levels did not differ among the experimental groups. Urinary flow increased, whereas urinary creatinine concentration decreased in the subAKI + Dapa and Dapa groups compared with the CTL group.

**TABLE 1:** Functional parameters (Mean ± SD).

Parameters	CTL (*n* = 14)	subAKI (*n* = 14)	subAKI + Dapa (*n* = 14)	Dapa (*n* = 8)
Body weight (g)	20.80 ± 1.26	21.07 ± 1.62	21.27 ± 1.10	23.13 ± 1.45
Kidney weight/Body weight (mg/g)	7.25 ± 0.10	7.78 ± 1.31	7.80 ± 0.09	7.76 ± 0.08
Water intake (mL/24 h)	5.0 ± 1.60	4.46 ± 1.44	4.85 ± 1.48	4.43 ± 0.56
Food intake (g/24 h)	3.81 ± 0.36	3.64 ± 0.84	3.92 ± 0.61	4.40 ± 0.49^a^
Sodium intake (mg/24 h)	10.31 ± 0.95	10.18 ± 1.88	10.61 ± 1.66	11.96 ± 1.33^a^
Plasma sodium (mmol/L)	148 ± 24.87	138.8 ± 16.70	150.8 ± 21.77	134.2 ± 9.02
Urinary flow (μL/min)	0.62 ± 0.19	0.87 ± 0.15	1.06 ± 0.32^a^	1.12 ± 0.28^b^
Urinary creatinine (mg/dL)	73.01 ± 13.65	62.95 ± 10.98	51.62 ± 11.85^b^	44.17 ± 8.37^c^

^a^ Compared to CTL.

^b^ Compared to subAKI;

^c^ p < 0.05.

**FIGURE 1:**
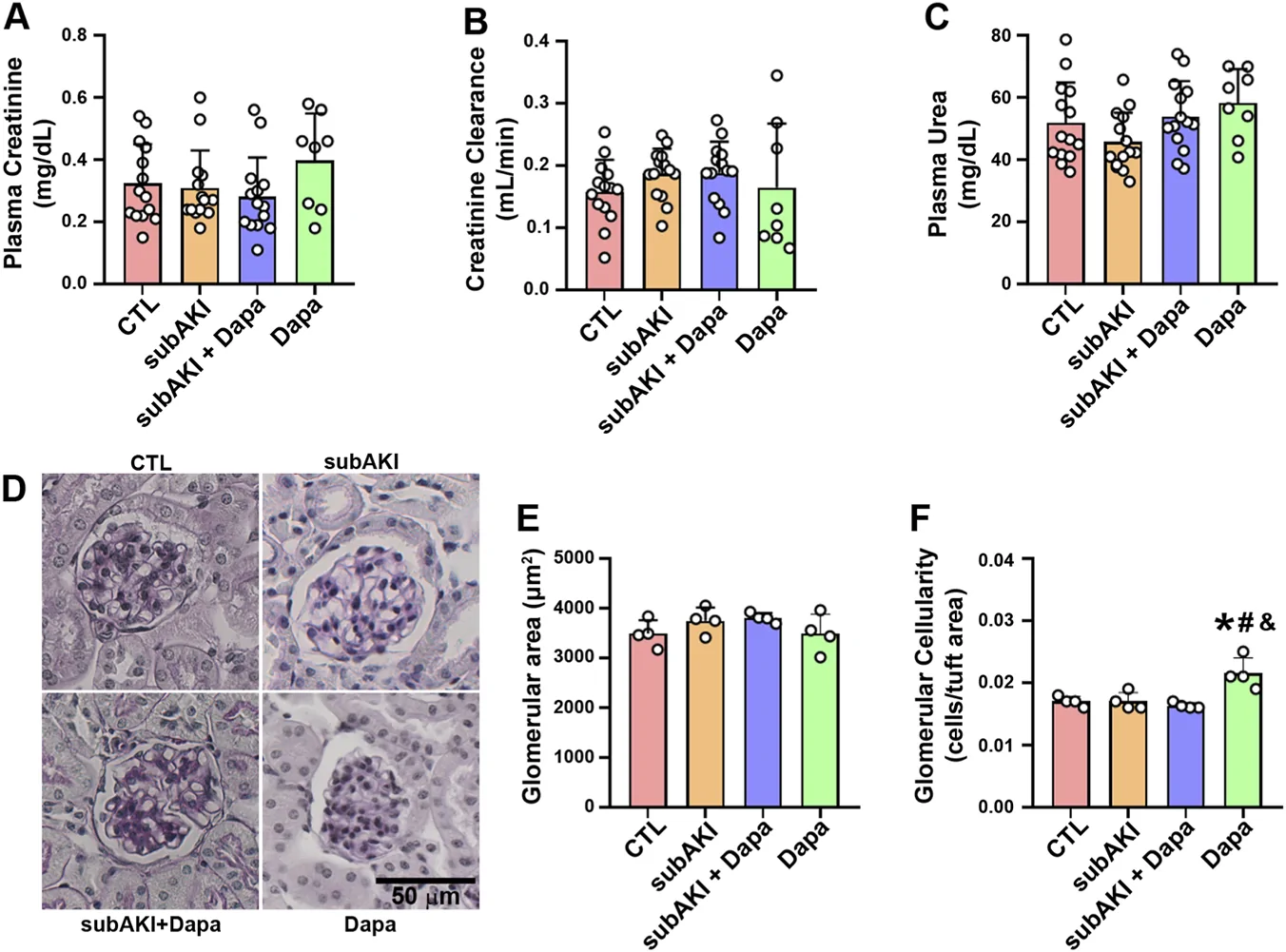
Dapagliflozin treatment on glomerular structure and function. **(A)** Plasma creatinine. **(B)** Creatinine clearance. **(C)** Plasma urea. **(D)** Representative photomicrographs of cortical glomeruli in kidney sections stained with periodic acid-Schiff, scale bar, 50 µm. **(E)** Quantitative analysis of glomerular area. **(F)** Glomerular cellularity. In panels E and F, each data point represents the mean value obtained from measurements of at least 10 different glomeruli (*n* = 4). Data are presented as mean ± SD. *compared to CTL; ^#^compared to subAKI; ^&^ compared to subAKI + Dapa; *^,#,&^ = p < 0.05.

The assessment of glomerular function parameters was performed based on plasma creatinine and urea, as well as eGFR determined by the creatinine clearance. Glomerular structure was assessed by analyzing glomerular tuft area and glomerular cellularity in kidney slices stained with periodic-acid Schiff (PAS). There was no change in these parameters among the experimental groups, except for the Dapa group, which presented increased glomerular cellularity (Figures 1A–F; Supplementary Figure S1).

**FIGURE 2:**
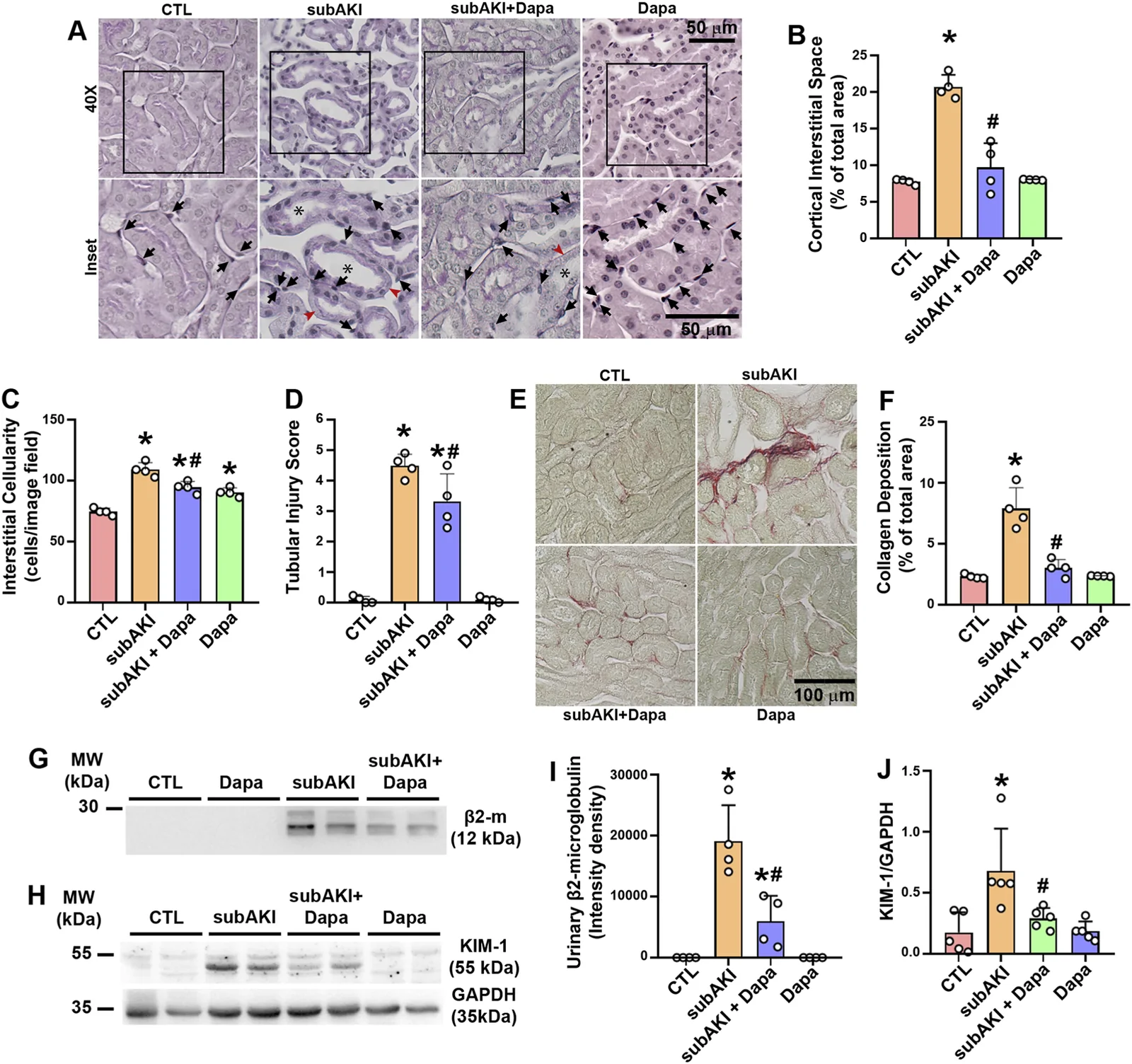
Dapagliflozin treatment attenuates tubule-interstitial injury markers observed in subclinical acute kidney injury. **(A)** Representative photomicrographs of cortical tubules in kidney sections stained with periodic acid-Schiff, scale bar, 50 µm. Black arrows indicate interstitial cells; red arrowheads indicate tubular atrophy; * shows tubular dilation. **(B)** Quantitative analysis of cortical interstitial space. **(C)** Interstitial cellularity. **(D)** Tubular injury score. **(E)** Representative photomicrographs of cortical tubules in kidney sections stained with Picrosirius red, scale bar, 100 µm. **(F)** Quantitative analysis of collagen deposition. **(G)** Representative immunoblot membrane of urinary β2-microglobulin (12 kDa). **(H)** Representative immunoblot membrane of KIM-1 expression in kidney cortex (55 kDa). **(I)** Quantitative analysis of urinary β2-microglobulin. **(J)** Quantitative analysis of KIM-1 expression in relation to GAPDH used as loading control. In panels B, C, D, and F each data point represents the mean value obtained from measurements of at least 10 different image fields (*n* = 4). Data are presented as mean ± SD. *compared to CTL; ^#^compared to subAKI; *^,#^ = p < 0.05.

To analyze the effect of Dapa on the development of subAKI-induced TII, we measured both histological and functional tubular damage biomarkers. It was assessed by cortical tubule-interstitial space, cortical interstitial cellularity, tubular injury score, and collagen deposition as well as urinary β2M and cortical expression of KIM-1 (Figures 2A–J; Supplementary Figure S2). All parameters were increased in the subAKI group compared to the CTL group while simultaneous Dapa treatment (subAKI + Dapa) attenuated these effects (Figures 2A–J). Regarding the effects of dapagliflozin in healthy animals, no significant changes were observed in the functional or histological markers of TII, except for interstitial cellularity. Specifically, the Dapa group exhibited a significant increase in interstitial cellularity compared with the CTL group.

Together, these results showed that dapagliflozin treatment ameliorates TII observed in subAKI. Next, we investigated the effects of dapagliflozin on renal solute handling during subAKI development.

### 3.2 Proteinuria and albuminuria are attenuated by dapagliflozin treatment in subclinical acute kidney injury

**FIGURE 3:**
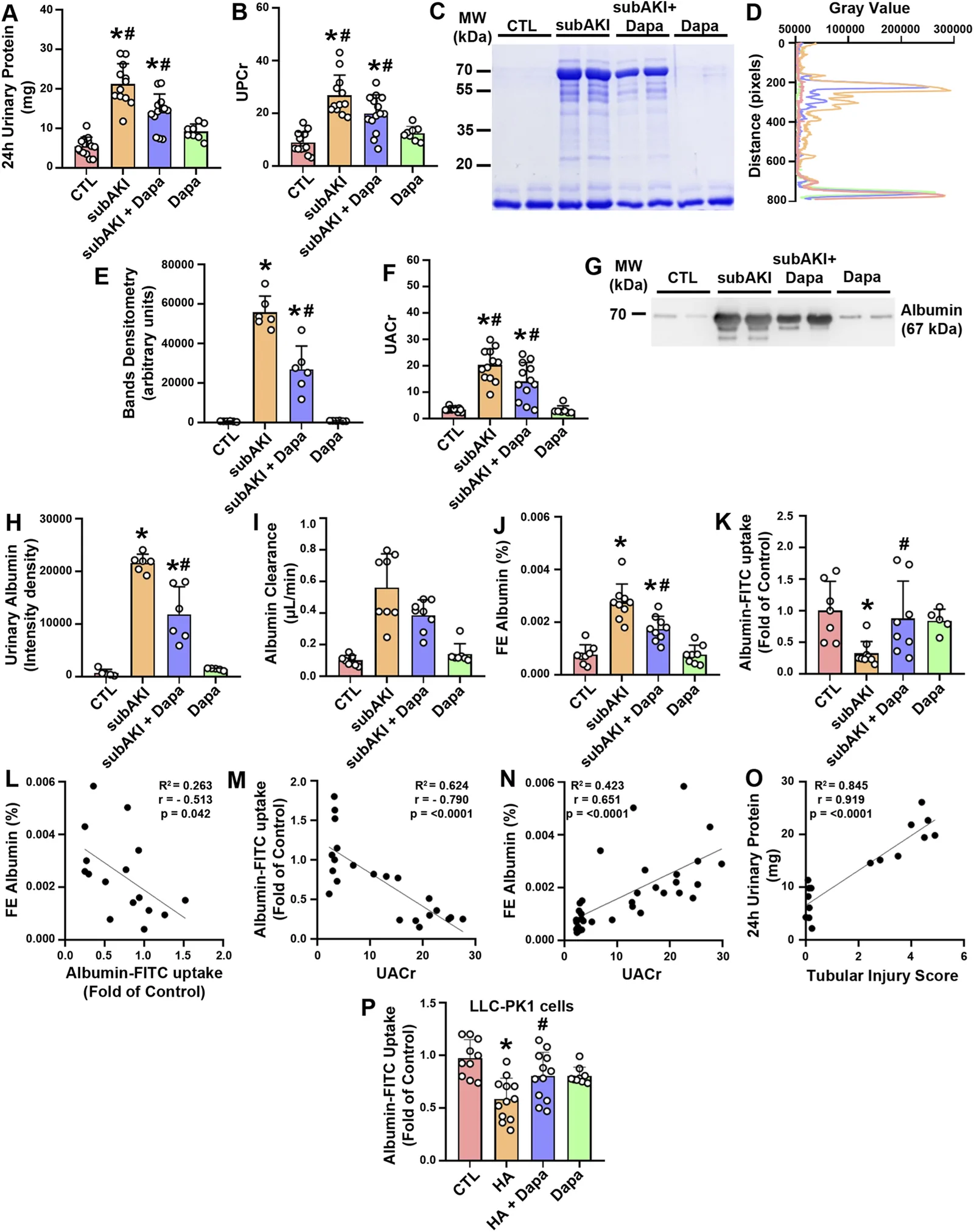
Dapagliflozin treatment attenuates proteinuria and albuminuria in subclinical acute kidney injury. **(A)** 24 h Urinary protein. **(B)** Urinary protein-to-creatinine ratio (UPCr). **(C)** SDS-PAGE analysis of urinary proteins stained with Coomassie blue. **(D)** Plot profile analysis of band intensity. **(E)** Densitometry analysis of urinary protein (∼70 kDa bands) from SDS-PAGE. **(F)** Urinary albumin-to-creatinine ratio (UACr). **(G)** Representative immunoblot membrane of urinary albumin (67 kDa). **(H)** Quantitative analysis of urinary albumin. **(I)** Albumin clearance (µL/min). **(J)** Renal fractional excretion of albumin (FEalbumin). **(K)** Cortical albumin-FITC uptake *in vivo*. **(L)** Pearson’s correlation between FEalbumin and albumin-FITC uptake. **(M)** Pearson’s correlation between albumin-FITC uptake and UACr. **(N)** Pearson’s correlation between FEalbumin and UACr. **(O)** Pearson’s correlation between 24 h urinary protein and tubular injury score. **(P)** Albumin-FITC uptake *in vitro* (*n* represents individual wells in at least three independent experiments). HA: high albumin (20 mg/mL); HA + Dapa: high albumin plus dapagliflozin (5 µM). Pearson’s correlation analyses were performed using pooled data from all experimental groups. Data are presented as mean ± SD. *compared to CTL; ^#^compared to subAKI or HA; *^,#^ = p < 0.05.

Since proteinuria and albuminuria are hallmarks of the subAKI condition, we investigated the effects of Dapa on these parameters. Twenty-four hour (24 h) proteinuria and the urinary protein-to-creatinine ratio (UPCr) were increased in the subAKI group compared with the CTL group (Figures 3A,B). In addition, the subAKI group exhibited increased low-to middle-molecular weight proteinuria, assessed by SDS-PAGE, along with higher urinary albumin-to-creatinine ratio (UACr) and urinary albumin excretion measured by immunoblotting (Figures 3C–H). A rabbit monoclonal anti-albumin antibody [EPR20195], which specifically recognizes mouse, rat, and human albumin but does not detect bovine albumin, was used for immunoblotting analysis. Albumin handling was further evaluated by measuring albumin clearance and urinary fractional excretion of albumin (FEalbumin) (Figures 3I,J), both of which were elevated in the subAKI group. Notably, Dapa treatment (subAKI + Dapa) attenuated all these alterations (Figures 3A–J).

To examine whether increased albuminuria was associated with impaired albumin reabsorption in PTECs, we assessed *in vivo* albumin uptake (Figure 3K). The subAKI group showed reduced PTEC albumin uptake compared with the CTL group, an effect that was prevented by dapagliflozin treatment.

The potential relationships among urinary albumin excretion, PTECs albumin reabsorption, and tubular injury were analyzed using Pearson’s correlation coefficient (Figures 3L–O). Data from all experimental groups were pooled to increase the range of values for each parameter and thereby assess the overall relationship between these variables. We observed a significant inverse correlation between FEalbumin and *in vivo* albumin-FITC uptake (Figure 3L), as well as between albumin-FITC uptake and UACr (Figure 3M). In contrast, a positive linear correlation was found between FEalbumin and UACr (Figure 3N), and between 24 h urinary protein excretion and tubular injury score (Figure 3O).

To evaluate the direct effect of Dapa treatment on albumin reabsorption in PTECs, we used LLC-PK1 cells, a well-established porcine PTEC line. To mimic albumin overload during subAKI development, LLC-PK1 cells were incubated overnight with a high albumin concentration (20 mg/mL). Under these conditions, albumin endocytosis was reduced in LLC-PK1 cells (Figure 3P). Notably, this effect was abolished when the cells were co-incubated with 5 μM dapagliflozin, consistent with the effect of dapagliflozin observed *in vivo* (Figure 3K).

These results indicate that dapagliflozin treatment attenuates the reduction in PTECs albumin reabsorption and, consequently, the increase in albuminuria observed in subAKI. However, whether this effect is specific to albumin transport or reflects a broader effect on solute transport in PTECs remains unclear. To address this question, we evaluated the effects of dapagliflozin on glucose and sodium handling in PTECs.

**FIGURE 4:**
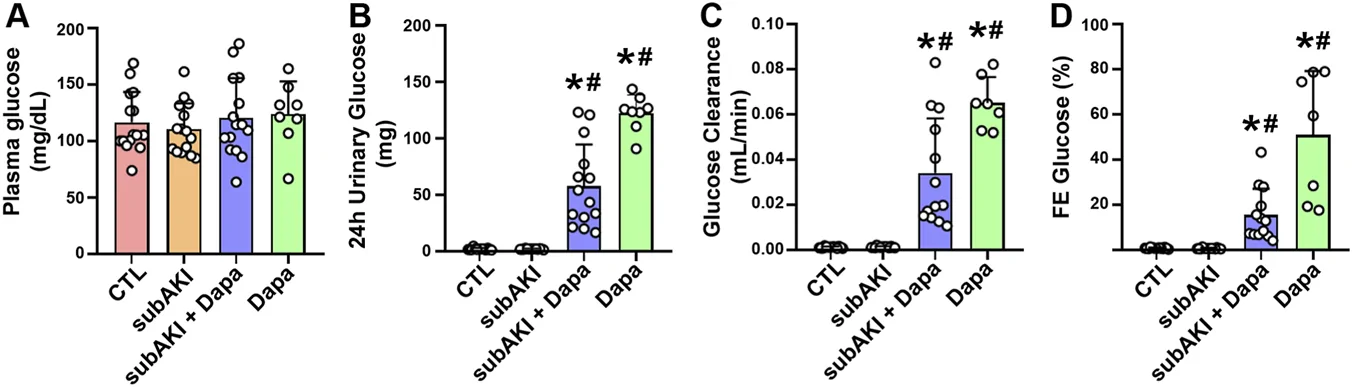
The effect of dapagliflozin treatment on plasma glucose and renal handling of glucose. **(A)** Plasma glucose. **(B)** 24 h Urinary glucose. **(C)** Glucose clearance (mL/min). **(D)** Renal fractional excretion of glucose (FEglucose). Data are presented as mean ± SD. *compared to CTL; ^#^compared to subAKI; *^,#^ = p < 0.05.

Plasma glucose levels remained unchanged across all experimental groups (Figure 4A). However, 24 h urinary glucose excretion, glucose clearance, and urinary fractional excretion of glucose (FEglucose) were increased in the subAKI + Dapa and Dapa groups compared with both the CTL and subAKI groups (Figures 4B–D). These findings are consistent with the expected pharmacological effects of dapagliflozin on glucose transport.

**FIGURE 5:**
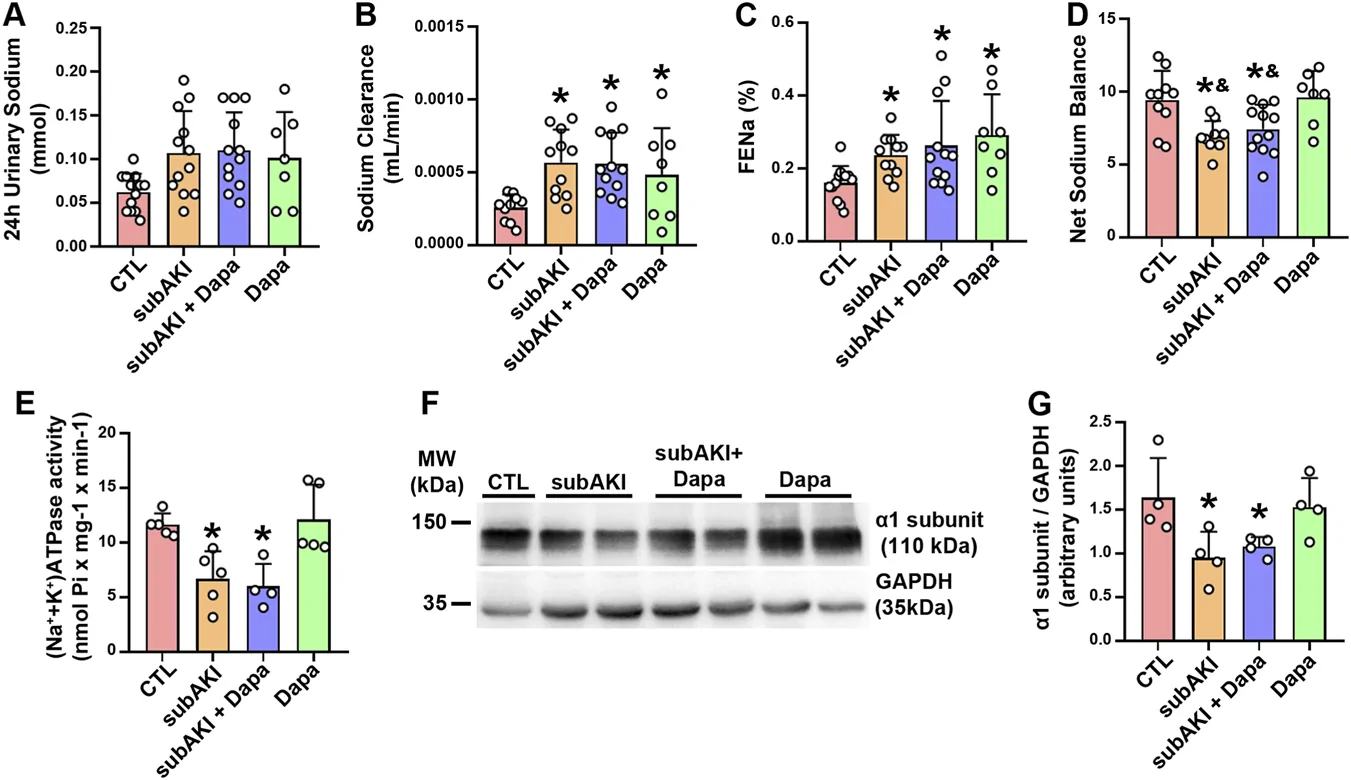
The effect of dapagliflozin treatment on renal handling of sodium and (Na^+^+K^+^)ATPase. **(A)** 24 h Urinary sodium. **(B)** Sodium clearance. **(C)** Renal fractional excretion of sodium (FE_Na_). **(D)** Net sodium balance. **(E)** (Na^+^+K^+^)ATPase activity in renal cortex (*n* = 5). **(F)** Representative immunoblot membrane of cortical α1 subunit of (Na^+^+K^+^)ATPase (110 kDa). **(G)** Quantitative analysis of α1 subunit expression in relation to GAPDH used as loading control. Data are presented as mean ± SD. *compared to CTL; ^#^compared to subAKI; ^&^compared to Dapa. *^,#,&^ = p < 0.05.

Assessment of urinary sodium handling revealed that the subAKI, subAKI + Dapa, and Dapa groups exhibited increased 24 h urinary sodium excretion, sodium clearance, and urinary fractional excretion of sodium (FENa) (Figures 5A–C). Net sodium balance was reduced in the subAKI and subAKI + Dapa groups (Figure 5D). Similarly, cortical (Na^+^+K^+^)ATPase activity and expression were decreased in the subAKI and subAKI + Dapa groups (Figures 5E,F). Dapagliflozin treatment alone did not affect net sodium balance or (Na^+^+K^+^)ATPase activity and expression.

Taken together, these results indicate that dapagliflozin specifically attenuates the impairment in protein reabsorption, but not the reduction in sodium reabsorption, in PTECs during subAKI. This selective effect on protein handling in PTECs appears to be associated with the development of tubule-interstitial injury.

### 3.3 Cortical protein *O*-GlcNAcylation is modulated by dapagliflozin treatment in subclinical acute kidney injury

**FIGURE 6:**
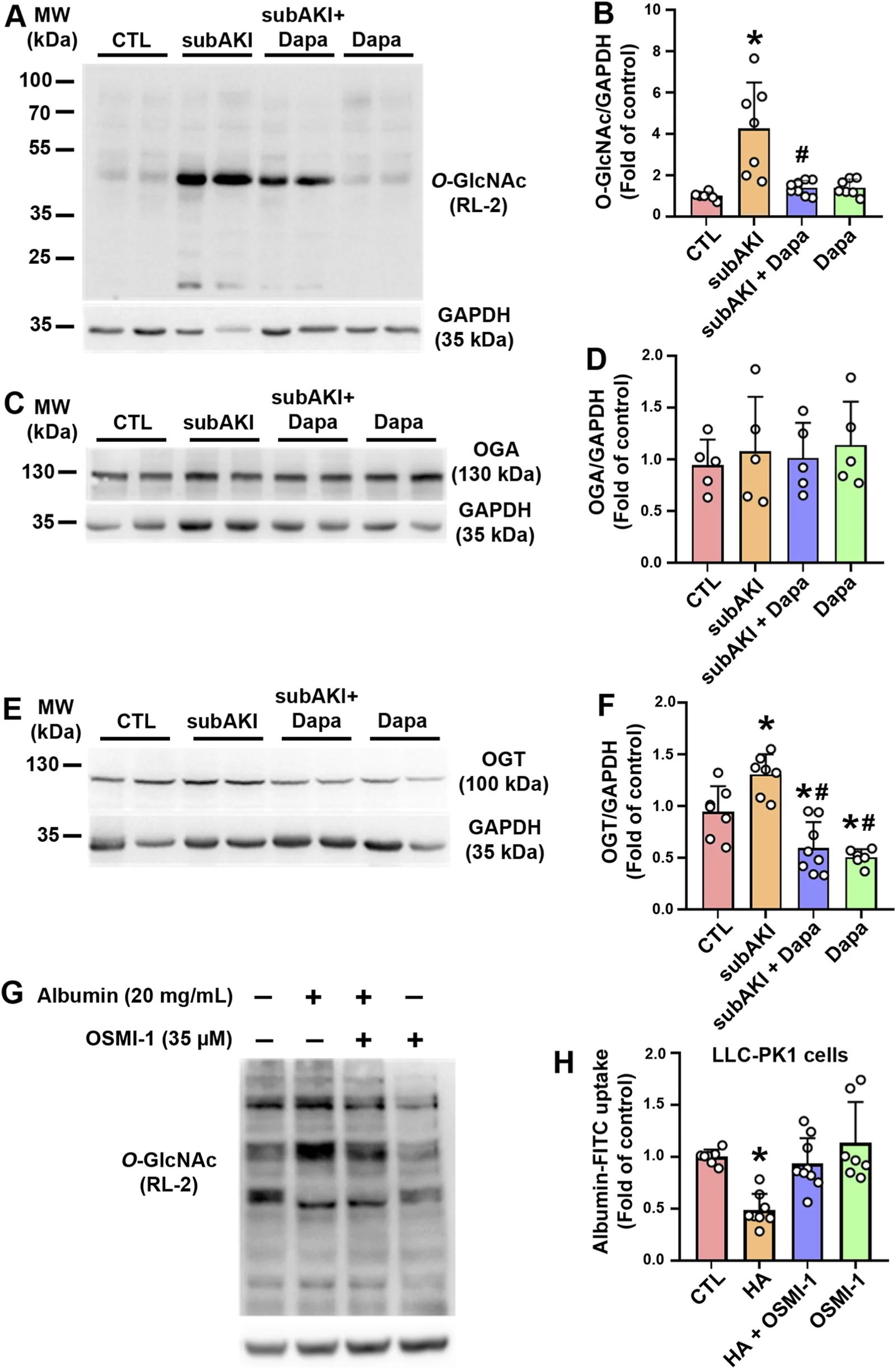
Kidney cortex protein O-GlcNAcylation is modulated by dapagliflozin treatment in subclinical acute kidney injury. **(A)** Representative immunoblot membrane of total protein *O-*GlcNAc expression in kidney cortex. **(B)** Quantitative analysis of total protein *O*-GlcNAc expression in relation to GAPDH used as loading control, the entire lane was used for densitometric analysis. **(C)** Representative immunoblot membrane of OGA expression in kidney cortex (130 kDa). **(D)** Quantitative analysis of OGA expression in relation to GAPDH used as loading control. **(E)** Representative immunoblot membrane of OGT expression in kidney cortex (100 kDa). **(F)** Quantitative analysis of OGT expression in relation to GAPDH used as loading control. **(G)** Representative immunoblot membrane of total protein *O-*GlcNAc in LLC-PK1 cells under high albumin concentration (20 mg/mL) in the presence or absence of OSMI-1 (35 µM). **(H)** Albumin-FITC uptake in LLC-PK1 cells under high albumin concentration (20 mg/mL) in the presence or absence of OSMI-1 (35 µM) (*n* represents individual wells in at least three independent experiments). HA: high albumin (20 mg/mL); OSMI-1: cell permeable inhibitor of OGT. HA + OSMI-1: high albumin plus OSMI-1 (35 µM). Data are presented as mean ± SD. *compared to CTL; ^#^compared to subAKI; *^,#^ = p < 0.05.

To further explore the underlying mechanisms, we investigated if dapagliflozin could modulate *O*-GlcNAcylation. Total protein *O*-GlcNAcylation in renal cortex was increased in subAKI group in comparison to the CTL group which was attenuated by Dapa treatment (subAKI + Dapa, Figures 6A,B). Given that the balance between OGA and OGT may determine the status of total protein *O*-GlcNAcylation, we also analyzed their expression. OGA expression was not modified among experimental groups (Figures 6C,D). On the other hand, OGT expression was increased in renal cortex homogenates from the subAKI group. Dapagliflozin treatment not only attenuated this increase but also reduced OGT expression below CTL levels in the subAKI + Dapa group (Figures 6E,F). A similar effect was observed in the Dapa group. Together, these results suggest that the protective effects of dapagliflozin are associated with reduced OGT expression, thereby preventing the subAKI-induced increase in total protein O-GlcNAcylation.

To gain further insight into the role of OGT in the relationship between *O*-GlcNAcylation levels and albumin uptake, LLC-PK1 cells were treated with OSMI-1, a cell-permeable inhibitor of OGT. Consistent with findings from the subAKI mouse model, exposure to a high albumin concentration increased total protein *O*-GlcNAcylation while reducing albumin uptake in LLC-PK1 cells (Figures 6G,H). Notably, OSMI-1 prevented the effects of high albumin on both total protein *O*-GlcNAcylation and albumin uptake in LLC-PK1 cells. Taken together, these results suggest that the effects of dapagliflozin on protein reabsorption in PTECs during subAKI development may involve modulation of OGT-dependent O-GlcNAcylation.

## 4 Discussion

Subclinical acute kidney injury remains an underrecognized syndrome with limited therapeutic options, placing patients at higher risk for the development of AKI and CKD (Haase et al., 2012; Dépret et al., 2020). Dysfunction in protein reabsorption by PTECs contributes to the pathogenesis of subAKI, making this process a potential therapeutic target to prevent the development of TII (Peruchetti et al., 2021; Peres et al., 2023a). In the present study, we showed that simultaneous dapagliflozin treatment with induction of subAKI animal model ameliorates TII development, which was associated with decreased cortical protein *O*-GlcNAcylation, leading to an anti-proteinuric effect.

In the present work, a well-known subAKI mouse model based on the daily peritoneal injection of BSA was used (Ishola et al., 2006; Portella et al., 2013; Peruchetti et al., 2021; Peres et al., 2023a). In this animal model, TII develops as a consequence of albumin overload in PTECs, resulting from increased glomerular filtration of albumin associated or not with minimal injury process. In a previous study, our group demonstrated in SV129 mice that nestin expression was increased in the subAKI model and was associated with increased glomerular tuft surface density and mesangial surface density, indicating an expansion of the glomerular filtration surface area. These structural changes are consistent with the observed increase in glomerular albumin filtration (Landgraf et al., 2014). Despite these alterations, no changes in creatinine clearance or in plasma creatinine levels were detected. Likewise, Eddy et al. (2000) reported that B6SJL mice injected with BSA for 10 days showed no significant alterations in the glomerular filtration barrier, as assessed by electron microscopy, or in plasma creatinine levels. Ishola et al. (2006) using a model of albumin overload in C57BL/6J and 129S2/Sv mouse also observed, using electron microscopy, no remarkable structural variations in the glomerular filtration barrier associated with no changes in eGFR.

Herein, histological parameters and creatinine clearance were used to determine glomerular structure and eGFR showing that these parameters were not changed. Similarly, no significant modifications in eGFR have also been reported by our group in previous studies (Portella et al., 2013; Peruchetti et al., 2021; Peres et al., 2023a). Our results showed albuminuria and proteinuria associated with an increase in functional and structural markers of TII. Together, the results obtained in the present work with those obtained in previous studies showed that the mouse model used in the present work represents a reliable and appropriate model for studying the subAKI syndrome.

SubAKI represents a spectrum of different etiologies characterized by no changes in plasma creatinine and/or urinary flow associated with presence of tubular damage biomarkers (Haase et al., 2012). Therefore, the findings observed in the present manuscript could not be reproduced in all clinical subAKI conditions. Another potential limitation of the present study is that only male mice were included, precluding the assessment of possible sex-specific effects of dapagliflozin. Sex-related differences in the physiology of PTECs have been previously reported (Sabolić et al., 2007). Therefore, future studies including both sexes will be important to determine whether the protective effects of dapagliflozin are generalizable across sexes.

It is worth mentioning that dapagliflozin treatment was initiated simultaneously with BSA administration, allowing us to evaluate whether the SGLT2 cotransporter is involved in the pathogenesis of subAKI. Our results demonstrate that dapagliflozin treatment, administered concurrently with the induction of the subAKI mouse model, attenuated proteinuria and albuminuria and was associated with reduced development of TII, indicating that SGLT2 may represent a potential therapeutic target for subAKI to prevent the progression of kidney disease. Usually, the anti-proteinuric effect of SGLT2i in CKD is associated with changes in glomerular protein filtration (Vallon and Thomson, 2017; Vallon and Thomson, 2020). Cassis et al. (2018), using a CKD animal model developed by unilateral nephrectomy associated with albumin injection, observed that the anti-proteinuric effect of dapagliflozin correlated with an improvement of podocyte injury. A similar effect was observed with dapagliflozin treatment in an aristolochic-acid (AA)-induced CKD mouse model (Oe et al., 2024). The beneficial effects of SGLT2i have also been demonstrated in other models of non-diabetic kidney disease, despite substantial differences in the underlying mechanisms (Hirashima et al., 2024; Oe et al., 2024; Zhao et al., 2023). However, the mechanism underlying the anti-proteinuric effect of gliflozins used was not completely understood. Herein, we showed in a subAKI mouse model as well as in LLC-PK1 cells, a porcine PTEC cell line, that the anti-proteinuric effect of dapagliflozin treatment is directly associated, at least in part, with modulation of PTECs albumin reabsorption. However, a potential effect of dapagliflozin on glomerular albumin filtration cannot be excluded, as this parameter was not evaluated in the present study.

Dapagliflozin has also demonstrated beneficial effects on additional renal parameters. In a randomized double-blind study involving patients with CKD and albuminuria, Russo et al. (2026) reported that dapagliflozin exerted a potential renoprotective effect, as evidenced by improved renal medullary oxygenation. Interestingly, this effect appeared to be more pronounced in nondiabetic patients. However, the authors did not discuss whether this effect was dependent on SGLT2 inhibition. It is also worth noting that the relationship between albuminuria and renal medullary oxygenation was not altered by dapagliflozin treatment, suggesting that these parameters may be regulated independently in CKD. Recently, Hu et al. (2024) demonstrated that dapagliflozin protected HK-2 cells from uric acid-induced fibrosis and mitochondrial dysfunction. Notably, these protective effects were preserved following CRISPR-Cas9-mediated knockout of SGLT2, indicating that dapagliflozin can exert SGLT2-independent effects in PTECs. In the present study, dapagliflozin produced the expected increase in urinary glucose excretion, confirming SGLT2 inhibition. However, we cannot exclude the possibility that some of its protective effects on the TII were mediated through SGLT2-independent mechanisms.

Hirashima and colleagues (2024) showed that luseogliflozin treatment attenuated glomerular and tubular damage biomarkers in a mouse model of ischemic AKI to CKD transition. We showed that dapagliflozin treatment also reduced the urinary level of tubular damage biomarkers, indicating that the anti-proteinuric effect of dapagliflozin could be correlated to TII development. Additionally, subAKI mouse model is well known for its reduced expression of megalin in the PTECs and impaired albumin-FITC uptake (Peruchetti et al., 2021; Peres et al., 2023a). In fact, we showed that dapagliflozin treatment restored albumin-FITC uptake in subAKI animals as well as abolished the inhibitory effect of high albumin concentration on the albumin endocytosis in LLC-PK1 cells. Additionally, Kapetanaki et al. (2022) showed that dapagliflozin also restored albumin uptake in HK-2 cells treated with trimethylamine N-oxide, a uremic toxin.

Based on this evidence, it is plausible that the protective effect of dapagliflozin on TII development in the subAKI mouse model involves modulation of the receptor-mediated albumin endocytic machinery. Consistent with this hypothesis, we previously demonstrated that dapagliflozin attenuates the high glucose-induced reduction in megalin expression in a mouse model of early diabetic kidney disease (Farias et al., 2023). However, other receptors, including the neonatal Fc receptor (FcRn), cubilin, and amnionless, also contribute to receptor-mediated albumin endocytosis in proximal tubule epithelial cells (PTECs) (Christensen and Birn, 2002; Molitoris et al., 2022). Supporting this concept, cubilin expression has been associated with albumin-induced inflammatory responses in HK-2 cells (Yang et al., 2008) and with proteinuria in patients with preeclampsia (Albejante et al., 2020). Therefore, although our findings support a role for preserved megalin expression in the dapagliflozin-mediated attenuation of albuminuria in subAKI, further studies are needed to define the relative contributions of megalin and other components of the albumin endocytic machinery.

We observed that the mechanism involved in dapagliflozin effects on the receptor-mediated albumin endocytosis in PTECs seems to involve changes in *O*-GlcNAcylation levels. It was observed that the inhibition of albumin uptake in PTECs is associated with increased *O*-GlcNAcylation. In agreement, two independent works also showed that specific SGLT2i reduced kidney *O*-GlcNAcylation in both STZ-induced and Akita diabetic models (Hodrea et al., 2020; Otomo et al., 2020). Furthermore, it was shown that phlorizin, a non-specific sodium-glucose co-transporter, could prevent high-glucose-induced *O*-GlcNAcylation in LLC-PK1 cells, which was associated with improved albumin-FITC uptake (Peruchetti et al., 2018).

In a previous study, we demonstrated that 5 µM dapagliflozin reversed the inhibitory effects of high glucose on (Na^+^+K^+^)ATPase activity and α1-subunit expression (Silva-Aguiar et al., 2023), an effect associated with reduced *O*-GlcNAcylation of the α1-subunit. In the present study, using an albumin overload model in which extracellular glucose remained unchanged, dapagliflozin attenuated the increase in total *O*-GlcNAcylation but did not restore (Na^+^+K^+^)ATPase activity or α1-subunit expression. These contrasting findings likely reflect differences between the experimental models, suggesting that the mechanisms regulating *O*-GlcNAcylation and its effects on (Na^+^+K^+^)ATPase differ between high glucose- and albumin overload-induced injury. Further studies are needed to clarify these mechanisms.

Cellular *O*-GlcNAcylation status may respond to a variety of stress and metabolic conditions (Hart et al., 2007; Hart et al., 2011). As shown in the present work, high albumin concentration may also be an important trigger of increased *O*-GlcNAcylation in subAKI and LLC-PK1 cells. Our data suggest that this effect may be associated with increased OGT expression in the kidney cortex of subAKI animals. Hodrea et al. (2020) also showed that dapagliflozin could reduce OGT expression in kidney tissue of diabetic mice as well as in HK-2 under high glucose conditions.

Some works including those from our group have shown that megalin is a target of *O*-GlcNAcylation, which modulates its expression and function (Silva-Aguiar et al., 2018). Our group showed that increased *O*-GlcNAcylation of megalin results in reduced protein reabsorption in SHR rats and albumin-FITC uptake in LLC-PK1 cells (Silva-Aguiar et al., 2018). These data could suggest that anti-proteinuric effect of dapagliflozin could be correlated with direct megalin *O*-GlcNAcylation which could be involved in the development of TII. In accordance, our group have also shown that the development of TII in subAKI animal model involves a decrease in megalin expression (Peruchetti et al., 2021; Peres et al., 2023a). Interestingly, Long et al. (2022) showed that CRISPR/Cas9 knockout for megalin in OK cells, a model of PTECs, increased pro-inflammatory pathways and decreased PI3K/Akt pathway. Further experiments are necessary to confirm the direct *O*-GlcNAcylation of megalin induced by high albumin concentration and its role in the anti-proteinuric effect of dapagliflozin.

Albumin overload is well known for its pro-inflammatory and pro-fibrotic effects contributing to TII development in different kidney disease models, including subAKI (Tang et al., 2003; Peres et al., 2023a). The underlying mechanism involves the secretion of soluble factors by the PTECs, including cytokines and chemokines, thereby promoting immune cell infiltration into the renal interstitium (Tang et al., 2003; Lai et al., 2007; Fang et al., 2017). Several studies have highlighted the contribution of receptor-mediated albumin endocytosis to PTEC injury and kidney inflammation (Silva-Aguiar et al., 2018; Charlton et al., 2020; Peres et al., 2023a; Youm et al., 2024). Interestingly, the protective effects of SGLT2i have been attributed to their anti-inflammatory properties (Cai et al., 2023; Nogueira-Coelho et al., 2026). Indeed, dapagliflozin treatment attenuated kidney injury and alleviated renal inflammation (Cai et al., 2023). Moreover, empagliflozin treatment attenuated GFR decline along with a shift in renal macrophages from a pro-inflammatory to a resolutive phenotype (Nogueira-Coelho et al., 2026). While these studies suggest an anti-inflammatory effect, it is not clear if this is a direct effect of SGLT2i on immune cells, such as macrophages, or an indirect effect dependent on immunomodulatory factors derived from kidney cells (Cai et al., 2023; Nogueira-Coelho et al., 2026). Therefore, whether the protective effects of dapagliflozin observed in subAKI result from direct modulation of inflammatory pathways in PTECs or occur indirectly through restoration of receptor-mediated endocytosis remains an open question.

**FIGURE 7:**
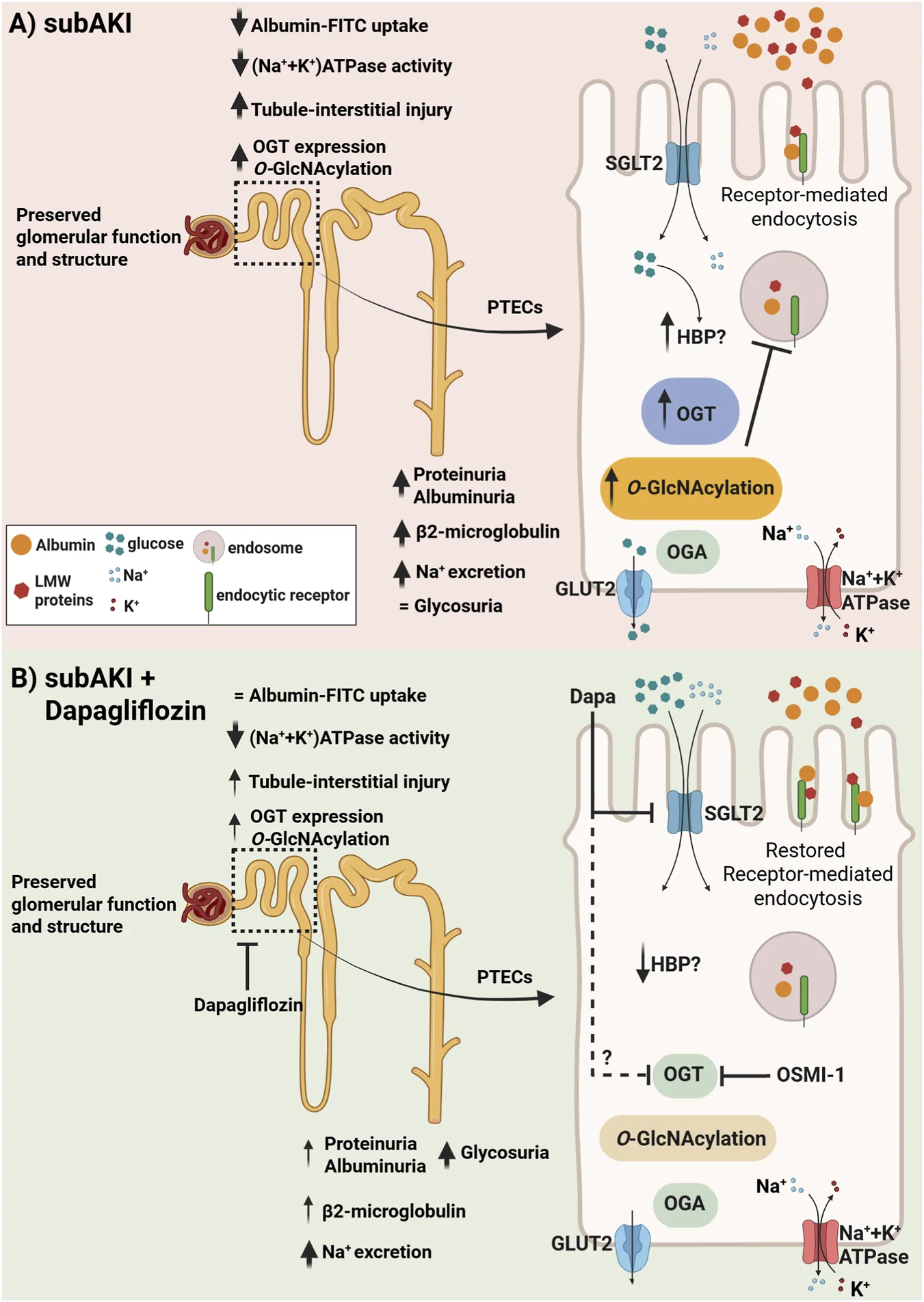
Proposed mechanisms underlying the effects of dapagliflozin in subAKI development. **(A)** Main findings observed in subAKI in response to albumin overload. Increased albumin levels in PTECs impair receptor-mediated albumin endocytosis, thereby exacerbating albuminuria and promoting tubule-interstitial injury. This phenotype is associated with increased OGT expression and total protein *O*-GlcNAcylation in the kidney cortex. Whether increased HBP activity contributes to these alterations remains to be elucidated. **(B)** Dapagliflozin co-treatment in subAKI restores albumin-FITC uptake in PTECs, thus attenuating albuminuria and tubule-interstitial injury parameters. These effects are associated with reduced OGT expression and decreased total protein *O*-GlcNAcylation, which may contribute to the restoration of receptor-mediated endocytosis and PTECs function. It remains unclear whether the effects of dapagliflozin on *O*-GlcNAcylation result from a shift of intracellular glucose away from the HBP or a direct effect on OGT expression. Direct pharmacological inhibition of OGT using OSMI-1 also improves albumin-FITC uptake in LLC-PK1 cells, similar to dapagliflozin. GLUT2, glucose transporter 2; HBP, hexosamine biosynthetic pathway; OGA, *O*-GlcNAcase; OGT, *O*-GlcNAc transferase; OSMI-1, cell permeable inhibitor of OGT; PTECs, proximal tubule epithelial cells; SGLT2, sodium-glucose cotransporter 2; subAKI, subclinical acute kidney injury. Symbols are shown in the lower left corner of the upper panel.

Together, our results suggest that the protective effect of dapagliflozin on TII development in subAKI mouse model is associated with the restoration of total protein *O*-GlcNAcylation balance, thereby contributing to its anti-proteinuric effect. The proposed mechanisms underlying the protective effects of dapagliflozin in subAKI are summarized in Figure 7. These data provide a strong rationale for future studies investigating the upstream signaling pathways involved in development of TII induced by albumin overload, as well as the potential application of dapagliflozin in the treatment of different subAKI clinical conditions.

## Data Availability

The raw data supporting the conclusions of this article will be made available by the authors, without undue reservation.
